# Maintenance Treatment of Newly Diagnosed Advanced Ovarian Cancer: Time for a Paradigm Shift?

**DOI:** 10.3390/cancers13225756

**Published:** 2021-11-17

**Authors:** Paul DiSilvestro, Nicoletta Colombo, Philipp Harter, Antonio González-Martín, Isabelle Ray-Coquard, Robert L. Coleman

**Affiliations:** 1 Program in Women’s Oncology, Women & Infants Hospital, Providence, RI 02905, USA; 2 IRCCS European Institute of Oncology (IEO), University of Milan-Bicocca, 20126 Milan, Italy; nicoletta.colombo@ieo.it; 3 Department of Gynecology & Gynecologic Oncology, Ev. Kliniken Essen Mitte, 45136 Essen, Germany; p.harter@kem-med.com; 4 Department of Oncology, Clínica Universidad de Navarra, 28027 Madrid, Spain; agonzalezma@unav.es; 5 Program in Solid Tumors, Center for Applied Medical Research (CIMA), 31008 Pamplona, Spain; 6 Centre Leon Berard, Claude Bernard, Claude Bernard University, 69008 Lyon, France; isabelle.ray-coquard@lyon.unicancer.fr; 7 US Oncology Research, The Woodlands, Houston, TX 77380, USA; Robert.Coleman@usoncology.com

**Keywords:** PARP inhibitor, ovarian cancer, BRCA mutation, homologous recombination deficiency

## Abstract

**Simple Summary:**

Advanced epithelial ovarian cancer has a poor prognosis, but targeted therapies have been developed and are providing new hope. We reviewed recent results with PARP inhibitors as treatment and/or maintenance therapy following chemotherapy in newly diagnosed advanced ovarian cancer. Data confirm the benefit of PARP inhibitors in this setting, especially in subgroups with genomic instability. We describe the implications of these trial results for clinical practice, focusing on the need for a personalized treatment approach based on biomarker profile and other factors, including tolerability, cost considerations, and physician and patient preference. We have developed a systemic treatment algorithm for newly diagnosed advanced ovarian cancer, intended as a tool for clinicians to aid decision making in their daily practice. We also consider areas of future research, such as exploring further options for patients whose disease relapses following PARP inhibitor treatment.

**Abstract:**

Recent data have demonstrated substantial efficacy with poly (ADP-ribose) polymerase (PARP) inhibitors as treatment and/or maintenance therapy in patients with newly diagnosed advanced epithelial ovarian cancer (EOC). Here, we review efficacy and safety results from four recent Phase III trials in newly diagnosed EOC: SOLO1 (olaparib), PAOLA-1 (olaparib in combination with bevacizumab), PRIMA (niraparib), and VELIA (veliparib). The implications of these data for current clinical practice and areas for future research are discussed, including ongoing studies of targeted agents in the newly diagnosed setting. Data from SOLO1, PAOLA-1, PRIMA, and VELIA confirm the benefit of PARP inhibitors (olaparib, niraparib, veliparib) for women with newly diagnosed EOC. The greatest benefit was seen in patients with a *BRCA1* and/or *BRCA2* mutation or in the homologous recombination deficiency (HRD)-test positive subgroup. These four well-conducted studies have generated practice-changing data. However, deciding how to apply these results in clinical practice is challenging, and substantial differences in trial design impede cross-trial comparisons. Recent PARP inhibitor approvals (olaparib, niraparib) in the newly diagnosed EOC setting have provided new maintenance treatment options for a broader patient population. The results of these studies call for personalized medicine based on biomarker profile and other factors, including tolerability, cost considerations, and physician and patient preference. Important areas for future research include appropriate use of both BRCA mutation and HRD testing to inform magnitude of PARP inhibitor benefit as well as exploring further options for patients who are HRD-test negative and for those who become PARP inhibitor resistant.

## 1. Introduction

Epithelial ovarian cancer (EOC) is often diagnosed at an advanced stage and, despite aggressive surgical management and chemotherapy, the overall survival rate is poor. Without maintenance therapy, approximately 70% of patients relapse within 3 years following initial treatment [1], and recurrent EOC is typically incurable. Curative intent as a goal of treatment, therefore, represents an unmet need in the newly diagnosed setting.

**Figure 1:**
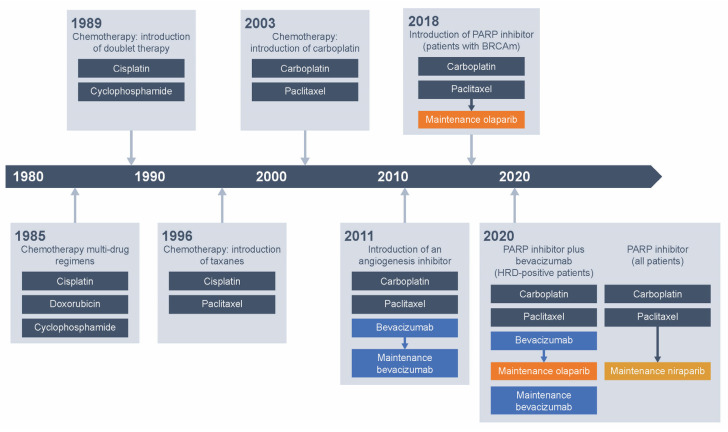
Development of standard systemic treatment for newly diagnosed advanced ovarian cancer. BRCAm, BRCA mutation; HRD, homologous recombination deficiency; PARP, poly (ADP-ribose) polymerase.

Until recently, the standard of care for newly diagnosed advanced EOC was surgery with carboplatin and paclitaxel chemotherapy ± bevacizumab followed by maintenance bevacizumab (Figure 1). Surgery should be performed prior to chemotherapy with the goal of macroscopic complete resection [2] or after neoadjuvant chemotherapy [3]. The antivascular endothelial growth factor (VEGF) monoclonal antibody bevacizumab was the first approved biological treatment for EOC.

The development of targeted therapies is providing new hope for treatment of EOC, with potential for cure. Here, we review recent results with poly (ADP-ribose) polymerase (PARP) inhibitors as treatment and/or maintenance therapy following platinum-based chemotherapy in newly diagnosed advanced EOC.

## 2. PARP Inhibitors: A New Standard of Care for Newly Diagnosed Advanced EOC

PARP inhibitors act by multiple mechanisms, including trapping PARP on DNA at sites of single-strand breaks, thereby preventing repair of the single-strand breaks and generating double-strand breaks during DNA replication that cannot be repaired accurately in tumors that have homologous recombination deficiency (HRD), such as tumors with a BRCA mutation (BRCAm, defined as a functional deficiency in *BRCA1* and/or *BRCA2*). PARP inhibitor treatment, therefore, leads to an accumulation of DNA damage and tumor cell death [4]. This is termed synthetic sickness (or synthetic lethality) because it requires two separate loss-of-function events, which are individually non-lethal, to occur simultaneously in order to result in tumor cell death. PARP inhibitors were developed with the intention of treating patients with HRD, specifically those with a BRCAm.

Several PARP inhibitors are being investigated in ovarian cancer clinical trials, including olaparib, rucaparib, niraparib, and veliparib. Early PARP inhibitor clinical studies in ovarian cancer focused on the recurrent setting. Maintenance treatment with PARP inhibitors has become a standard of care for recurrent EOC, with three PARP inhibitors approved in this setting. Olaparib [5,6], niraparib [7,8], and rucaparib [9,10] are approved in the USA and EU as maintenance treatment in women with platinum-sensitive relapsed EOC who are in response to their most recent platinum-based therapy, regardless of BRCAm status [5,6,7,8,9,10]. Additionally, olaparib is approved in the USA for patients with advanced EOC and a germline BRCAm who have received three or more prior lines of chemotherapy, regardless of sensitivity to platinum-based therapy [5]; niraparib is approved in the USA for patients with advanced EOC who have received three or more prior lines of chemotherapy and are HRD-positive (defined as BRCAm; or genomic instability and disease progression more than 6 months after response to last chemotherapy) [7]; and rucaparib is approved in the USA and EU for women with platinum-sensitive relapsed EOC who have a germline and/or somatic BRCAm and received at least two prior lines of chemotherapy (the EU approval specifies patients unable to tolerate further platinum-based chemotherapy) [9,10]. Given the potential long-term use of PARP inhibitors, understanding the tolerability profile of these drugs as maintenance therapy, both as a class effect and across individual safety profiles, is important when making treatment decisions [11].

All patients with newly diagnosed advanced EOC are at high risk of progression. SOLO1 was the first positive Phase III study of PARP inhibitor maintenance therapy following platinum-based chemotherapy for women with newly diagnosed advanced EOC and a BRCAm [12]. Three other PARP inhibitor Phase III studies in the first-line setting have recently published efficacy benefits in patients with or without BRCAm (Table 1). PAOLA-1 investigated maintenance olaparib combined with bevacizumab in a broad population with newly diagnosed stage III/IV high-grade ovarian cancer, irrespective of previous surgical outcome [13]. PRIMA investigated maintenance niraparib monotherapy and employed stricter inclusion criteria, e.g., excluding patients with stage III disease and no residual macroscopic disease after upfront surgery [14]. VELIA investigated veliparib combined with first-line chemotherapy followed by maintenance veliparib in newly diagnosed stage III/IV high-grade ovarian cancer [15]. Based on the wider activity of PARP inhibitors beyond patients with BRCAm in recurrent EOC [16,17,18], the trial populations included patients regardless of BRCAm. However, in all these trials, the greatest benefit with PARP inhibitors is seen in patients with BRCAm or HRD-test positive [13,14,15].

It should be noted that substantial differences in trial design in this setting impede cross-trial comparisons (Table 1). For example, VELIA calculated median progression-free survival (PFS) from the start of chemotherapy, whereas PAOLA-1 and PRIMA calculated PFS from the end of chemotherapy. PAOLA-1 was the only trial to include an active maintenance comparator with bevacizumab. One of the limitations of PRIMA and VELIA was the lack of a bevacizumab monotherapy arm, while a limitation of PAOLA-1 was the lack of a PARP inhibitor monotherapy arm. Additionally, the more stringent inclusion criteria in PRIMA means that these results may be less generalizable [14]. Improvements in upfront cytoreductive surgery mean that patients with complete resection may be increasingly represented within newly diagnosed patients [19,20]. Of the 61.9% of upfront surgery cases in SOLO1, 76.4% had no residual macroscopic disease [21].

These large Phase III studies all met their primary endpoints. SOLO1 data led to approval of olaparib for first-line maintenance treatment of BRCA-mutated advanced EOC in the USA, EU, Japan, and other countries, and mark a new era in the management of EOC (Figure 1; Table 2) [5,6,22]. Based on PAOLA-1 results, the US Food and Drug Administration (FDA) and the EU have expanded approval of olaparib in combination with bevacizumab to patients with advanced EOC and HRD-test positive status (defined by either a BRCAm and/or genomic instability) who are in complete or partial response to first-line platinum-based chemotherapy [5,6]. Based on PRIMA results, niraparib is approved for maintenance treatment of patients with advanced EOC who are in complete or partial response to first-line platinum-based chemotherapy [7].

### 2.1. SOLO1

**Figure 2:**
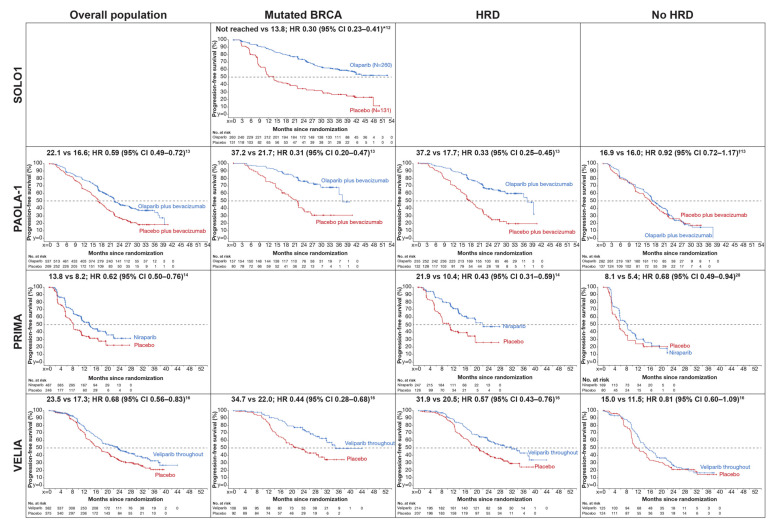
Summary of progression-free survival results from completed studies of poly (ADP-ribose) polymerase (PARP) inhibitors in newly diagnosed ovarian cancer. Data shown are median survival in months for experimental arm vs. control, HR, and 95% CIs. * The BRCA mutation population was the overall population for SOLO1. ^†^ The PAOLA-1 HRD-negative group also includes HRD unknown. The PRIMA Kaplan–Meier graph in the BRCA mutation population has not been published. CI, confidence interval; HR, hazard ratio; HRD, homologous recombination deficiency. Adapted from Refs [12,13,14,15,26]. SOLO1 [12] from *N. Engl. J. Med.*, Moore, K. et al. Maintenance olaparib in patients with newly diagnosed advanced ovarian cancer, Vol. 379, p2502. Copyright © (2018) Massachusetts Medical Society. Reprinted with permission from Massachusetts Medical Society. PAOLA-1 [13] from *N. Engl. J. Med.* Ray-Coquard, I. et al. Olaparib plus bevacizumab as first-line maintenance in ovarian cancer, Vol. 381, p2421, p2424 and Suppl. Appendix p19. Copyright © (2019) Mas-sachusetts Medical Society. Reprinted with permission from Massachusetts Medical Society. PRIMA overall population and HRD [14] from *N. Engl. J. Med.* González-Martín, A. et al. Niraparib in patients with newly diagnosed advanced ovarian cancer, Vol. 381, p2397. Copyright © (2019) Massachusetts Medical Society. Reprinted with permission from Massachusetts Medical Society. PRIMA No HRD [26] from European Medicines Agency (EMA). Assessment Report: Zejula. Available online: https://www.ema.europa.eu/en/documents/variation-report/zejula-h-c-003943-ii-0019-epar-assessment-report-variation_en.pdf. Copyright © European Medicines Agency, 2020. Reprinted with permission. VELIA 15 from *N. Engl. J. Med.*, Coleman, R.L. et al. Veliparib with first-line chemotherapy and as maintenance therapy in ovarian cancer, Vol. 381, p2410 and Suppl. Appendix p23. Copyright © (2019) Massachusetts Medical Society. Reprinted with permission from Massachusetts Medical Society.

SOLO1 evaluated olaparib maintenance therapy in 391 patients with newly diagnosed advanced EOC and a somatic or germline BRCAm, regardless of timing or outcome of surgery, who were in complete or partial response following platinum-based chemotherapy (Table 1) [12,21]. At the primary analysis, olaparib provided substantial improvement in the primary endpoint, investigator assessed PFS, with a 70% reduction in risk of disease progression or death versus placebo after a median follow-up of 41 months (Figure 2). With longer term follow-up, median PFS was 56.0 months with olaparib and 13.8 months with placebo, with 48.3% of olaparib patients versus 20.5% of placebo patients progression free at 5 years (Kaplan–Meier estimates) [23]. Despite pre-planned completion of olaparib therapy at 2 years in patients with a complete response or no evidence of disease, sustained benefit was seen beyond the end of treatment. At the primary analysis, a significant increase in time to second disease progression was also noted with olaparib (hazard ratio (HR) 0.50; 95% confidence interval (CI), 0.35–0.72; *p* < 0.001) [12]. Overall survival data are not yet mature but the permitted crossover to PARP inhibitors in the placebo arm may confound these findings (35% of patients who received subsequent therapy in the placebo arm used PARP inhibitors). No clinically meaningful change in health-related quality of life was seen within or between olaparib and placebo (Functional Assessment of Cancer Therapy—Ovarian Cancer Trial Outcome Index score) [12]. Exploratory patient-centered endpoints of quality-adjusted PFS and time without significant symptoms of toxicity (TWiST) demonstrated clinically meaningful benefits with olaparib versus placebo (*p* < 0.0001) [24].

Olaparib was generally well tolerated in SOLO1 (grade 3 or 4 adverse events (AEs), 39% vs. 18% with placebo; serious adverse events (SAEs), 21% vs. 12% with placebo) [12], with a safety profile consistent with previous studies [25]. The most common AEs of any-grade with olaparib were nausea (77% vs. 38% with placebo) and fatigue/asthenia (63% vs. 42% with placebo), and the most common grade 3/4 AEs were anemia (22% vs. 2% with placebo) and neutropenia (9% vs. 5% with placebo) (Table 3). Grade 3/4 thrombocytopenia occurred at low frequency in both arms (1% vs. 2% with placebo) (Table 3). Overall, the frequency of any-grade AEs leading to dose interruption was 52% and 17%, dose reduction was 28% and 3%, and discontinuation was 12% and 2%, in the olaparib and placebo arms, respectively. Information on acute myeloid leukemia (AML) and myelodysplastic syndrome (MDS) events were actively solicited during overall survival follow-up in the olaparib trials. At the primary analysis, AML occurred in 1% (3/260) of patients in the olaparib group and no patients in the placebo group [12]. Importantly, no new cases of MDS or AML were reported during longer term 5-year follow-up [23].

### 2.2. PAOLA-1

PAOLA-1 compared maintenance olaparib plus bevacizumab with placebo plus bevacizumab in 806 patients with newly diagnosed advanced EOC who had no evidence of disease or were in clinical complete or partial response after receiving chemotherapy plus bevacizumab, regardless of surgical status and BRCAm status [13] (Table 1). Bevacizumab represents an active standard of care in this setting, reporting PFS improvement and also OS benefit for some subgroups (high risk as defined by ICON7) [27]. Given the hypothesis that antiangiogenesis agents may cause hypoxia-induced HRD in the tumor [28] and potentially increase tumor sensitivity to olaparib, the bevacizumab and olaparib combination was of high interest. After 22.9 months’ median follow-up, median investigator assessed PFS (primary endpoint) was 22.1 vs. 16.6 months with olaparib vs. placebo (HR 0.59; 95% CI, 0.49–0.72; *p* < 0.001; Figure 2) [13].

Prespecified subgroup analyses showed that the greatest PFS benefit with olaparib vs. placebo was in tumor BRCA-mutated patients (median 37.2 vs. 21.7 months; HR 0.31; 95% CI, 0.20–0.47) [13]. PFS was also improved in HRD-test positive patients (myChoice^®^ (Myriad Genetics, Inc., Salt Lake City, UT, USA); HRD score ≥42), including BRCAm (median 37.2 vs. 17.7 months; HR 0.33; 95% CI, 0.25–0.45) and excluding BRCAm (median 28.1 vs. 16.6 months; HR 0.43; 95% CI, 0.28–0.66) [13]. A benefit was not seen with olaparib versus placebo in HRD-test negative or HRD status unknown patients (median 16.9 vs. 16.0 months; HR 0.92; 95% CI, 0.72–1.17; Figure 2) [13]. Median time until the first subsequent treatment for all patients was increased in the olaparib versus placebo group: 24.8 vs. 18.5 months (HR 0.59; 95% CI, 0.49–0.71) [13]. Overall survival data are immature [13]. Neither treatment group had a clinically significant change in health-related quality of life during the trial [13].

The median duration of treatment was 17.3 months (range 0–33) for olaparib and 15.6 months (range 0.1–26) for placebo [13]. The median duration of bevacizumab treatment since randomization was similar in both groups: 11.0 months (range 0.7–21) with olaparib and 10.6 months (range 0.7–17) with placebo [13]. AEs were consistent with the established safety profiles of olaparib and bevacizumab [13]. The most common AEs of all grades were fatigue/asthenia (53% vs. 32%), nausea (53% vs. 22%), and hypertension (46% vs. 60%) with olaparib plus bevacizumab versus placebo plus bevacizumab, respectively (Table 3) [13]. Hypertension is a frequent AE with bevacizumab, and the PAOLA-1 results showed that olaparib did not increase the rate of AEs associated with bevacizumab; indeed, the rate of hypertension was lower with olaparib/bevacizumab (46%) than with bevacizumab alone (60%) [13]. The incidence of grade ≥3 hematological AEs with olaparib/bevacizumab versus placebo/bevacizumab, respectively, was generally low: 17% vs. <1% for anemia, 7% vs. 1% for lymphopenia, 6% vs. 3% for neutropenia, 2% vs. 1% for leukopenia, and 2% vs. <1% for thrombocytopenia (Table 3) [13]. Overall, the frequency of grade ≥3 AEs was 57% and 51%, and the rate of any-grade AEs leading to dose interruption was 54% and 24%, dose reduction was 41% and 7%, and discontinuation was 20% and 6% with olaparib/bevacizumab and placebo/bevacizumab, respectively [13]. SAE incidence was 31% in both groups. MDS, AML, or aplastic anemia occurred in 1% (6/535) of patients receiving olaparib/bevacizumab and <1% (1/267) of patients receiving placebo/bevacizumab [13].

### 2.3. PRIMA

PRIMA investigated maintenance niraparib vs. placebo in 733 women with or without a BRCAm who had a complete or partial response following first-line platinum-based chemotherapy [14] (Table 1). The primary endpoint was PFS (by real-time blinded independent central review) in the overall population and HRD-test positive subgroup. After 13.8 months’ median follow-up, median PFS for niraparib vs. placebo was 13.8 vs. 8.2 months in the overall population (HR 0.62; 95% CI, 0.50–0.76; *p* < 0.001) and 21.9 vs. 10.4 months in HRD-test positive patients (HR 0.43; 95% CI, 0.31–0.59; *p* < 0.001) [14] (Figure 2). This benefit for niraparib vs. placebo was driven mainly by patients with a BRCAm (median PFS 22.1 vs. 10.9 months; HR 0.40; 95% CI, 0.27–0.62) and patients with HRD (myChoice^®^ HRD-test score ≥ 42) excluding BRCAm (median PFS 19.6 vs. 8.2 months; HR 0.50; 95% CI, 0.31–0.83) [14]. There was also an observed improvement in the HRD-test negative group, median PFS was 8.1 vs. 5.4 months for niraparib vs. placebo (HR 0.68; 95% CI, 0.49–0.94) [14] and in the HRD-unknown group, median PFS was 11.0 vs. 8.3 (HR 0.85; 95% CI, 0.51–1.43) [14,26]. Overall survival data were immature [14]. There was no evidence of a meaningful difference between groups in health-related quality of life [14].

The median duration of niraparib treatment was 11.1 months (range 0.03–29) [7]. Niraparib was associated with a higher incidence of AEs than with placebo, particularly hematological events. The most common AEs of any grade were thrombocytopenia (66% vs. 5%), anemia (64% vs. 18%), and nausea (57% vs. 28%) with niraparib vs. placebo, respectively (Table 2) [7]. The incidence of grade ≥3 hematological AEs with niraparib vs. placebo, respectively, was 39% vs. 0.4% for thrombocytopenia, 31% vs. 2% for anemia, 21% vs. 1% for neutropenia, and 5% vs. 0.4% for leukopenia (Table 3) [7]. The overall incidence of grade ≥3 AEs was 71% and 19%, SAE incidence was 32% and 13%, and the rate of any-grade AEs leading to dose interruption was 80% and 18%, dose reduction was 71% and 8%, and discontinuation was 12% and 2% in the niraparib and placebo arms, respectively [14]. The relatively high rates of dose interruption and reduction, as well as the low rate of discontinuation, suggest that AEs were managed by dose interruptions and reductions rather than discontinuations [14]. One case of MDS occurred in the niraparib group [14]. The PRIMA protocol was amended to change the dose from the approved fixed starting dose (FSD) of 300 mg once daily to an individualized starting dose (ISD; 200 or 300 mg once daily, depending on body weight and basal platelet count) [29]. PFS results were an HR of 0.59 (95% CI, 0.46–0.76) with FSD and an HR of 0.69 (95% CI, 0.48–0.98) with ISD [29]. The incidence of grade ≥3 hematological AEs was reduced with the ISD (thrombocytopenia 48% vs. 21%, anemia 36% vs. 22%, and neutropenia 24% vs. 15% with FSD vs. ISD, respectively) [8].

### 2.4. VELIA

VELIA incorporated a different treatment regimen to the studies described above by investigating veliparib in combination with platinum-based chemotherapy, followed by maintenance veliparib (Table 1) [15]. Patients with newly diagnosed stage III or IV high-grade serous ovarian carcinoma with and without a BRCAm were randomized evenly to three treatment arms (*n* = 1140): chemotherapy plus veliparib then veliparib maintenance (veliparib-throughout), chemotherapy plus veliparib then placebo maintenance (veliparib-combination only), or chemotherapy plus placebo then placebo maintenance (control). Median PFS was calculated from the start of chemotherapy, in contrast to the start of maintenance as in PRIMA, PAOLA-1, and SOLO1.

After 28 months’ median follow-up, investigator assessed PFS in the veliparib-throughout group (primary endpoint) was significantly prolonged vs. placebo in the BRCAm cohort (median 34.7 vs. 22.0 months, respectively; HR 0.44; 95% CI, 0.28–0.68; *p* < 0.001), the HRD-test positive cohort (myChoice^®^ HRD test score ≥ 33 or BRCAm) (HR 0.57; 95% CI, 0.43–0.76; *p* < 0.001), and the overall intent-to-treat population (HR 0.68; 95% CI, 0.56–0.83; *p* < 0.001), listed in order of testing hierarchy (Figure 2) [15]. However, the independent value of adding veliparib during induction therapy without veliparib maintenance was less clear; median PFS was similar in the veliparib-combination-only arm compared with control (BRCAm cohort median 21.1 vs. 22.0 months (HR 1.22; 95% CI, 0.82–1.80), HRD-test positive cohort median 18.1 vs. 20.5 months (HR 1.10; 95% CI, 0.86–1.41), and overall population median 15.2 vs. 17.3 months (HR 1.07; 95% CI, 0.90–1.29)) [15].

Additionally, the absence of a comparator arm with veliparib in the maintenance phase makes it difficult to determine the relative contributions of concurrent and maintenance veliparib therapy in the veliparib-throughout group. Exploratory PFS analyses compared veliparib-throughout vs. placebo in a HRD-test negative subgroup (median 15.0 vs. 11.5 months; HR 0.81; 95% CI, 0.60–1.09) and a non-BRCAm subgroup (including HRD-test negative and some HRD-test positive tumors; median 18.2 vs. 15.1 months; HR 0.80; 95% CI, 0.64–1.00) [15]. In all groups, disease-related symptom scores improved, particularly after chemotherapy was completed [15]. There were no clinically significant differences between the treatment groups [15].

Veliparib added to chemotherapy (veliparib throughout) led to a higher incidence of gastrointestinal and hematological AEs in this study vs. chemotherapy alone [15]. During the maintenance phase, the most common AEs were nausea (56% vs. 24%) and vomiting (34% vs. 12%) for veliparib-throughout vs. control, and grade 3/4 hematological AEs were generally low: 5% vs. 4% for neutropenia, 7% vs. 1% for anemia, 7% vs. <1% for thrombocytopenia, and 1% in both groups for leukopenia (Table 2) [15]. The overall frequency of grade 3/4 AEs in the maintenance phase was 45% and 32%, and the rate of AEs leading to veliparib/placebo dose interruption was 41% and 19%, veliparib/placebo dose reduction was 24% and 4%, and veliparib/placebo discontinuation was 19% and 6% in the veliparib-throughout and control arms, respectively [15]. One case of MDS was reported in the veliparib-combination-only group and one case of AML was reported in the veliparib-throughout group [15].

## 3. Ongoing Trials in the Newly Diagnosed Setting

A number of studies are ongoing in the newly diagnosed setting and may reveal new therapeutic strategies. Several Phase III studies are investigating immuno-oncology agents (antiprogrammed cell death ligand 1 (PD-L1) agents and programmed cell death-1 receptor (PD-1) agents) in different combinations with chemotherapy and as maintenance therapy, including combinations with bevacizumab and/or PARP inhibitors. Two trials (IMaGYN050 and JAVELIN OVARIAN 100) evaluating the addition of anti-PD-L1 agents to platinum-based chemotherapy with or without bevacizumab failed to meet their primary endpoint of improvement in PFS. IMagyn050 (NCT03038100) [30] assessed atezolizumab added to carboplatin/paclitaxel with concurrent and maintenance bevacizumab and the JAVELIN OVARIAN 100 study (NCT02718417) [31] assessed avelumab combined with and/or following platinum-based chemotherapy. JAVELIN OVARIAN 100 has been terminated [31], whereas IMaGYN050 follow-up will continue to the next planned analysis [30]. The separate JAVELIN OVARIAN PARP 100 study (NCT03642132) aimed to assess avelumab combined with chemotherapy followed by maintenance avelumab plus talazoparib but was also discontinued [31], partly due to the JAVELIN OVARIAN 100 interim results.

Further studies investigating various combinations and treatment strategies are ongoing. ATHENA/ENGOT Ov45 (NCT03522246) [32] is evaluating the anti-PD-1 agent nivolumab in combination with rucaparib as maintenance therapy in patients with a response to first-line surgery/platinum chemotherapy. DUO-O/ENGOT Ov46 (NCT03737643) [33] is investigating several regimens containing the anti-PD-L1 agent durvalumab, including durvalumab in combination with chemotherapy plus bevacizumab followed by maintenance regimens comprising durvalumab and bevacizumab with or without olaparib. This study involves one cohort of patients with BRCA mutation (single arm) and one cohort without BRCA mutation (three arms). MK-7339-001/ENGOT Ov43 (NCT03740165) [34] is investigating the anti-PD-1 agent pembrolizumab combined with carboplatin/paclitaxel followed by maintenance olaparib in non-BRCA-mutated patients; concurrent and maintenance bevacizumab is optional. FIRST/ENGOT Ov44 (NCT03602859) [35] is assessing the anti-PD-L1 agent dostarlimab in combination with first-line paclitaxel/carboplatin, followed by maintenance dostarlimab plus niraparib; concurrent and maintenance bevacizumab is optional.

## 4. Implications for Clinical Practice

**Figure 3:**
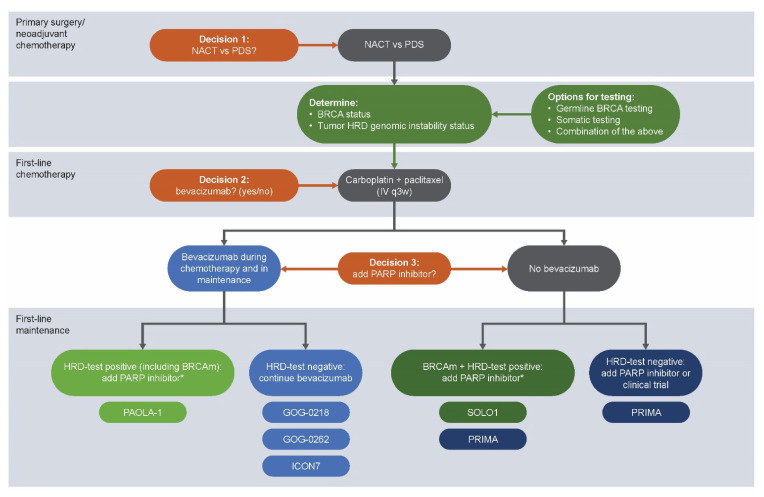
Proposed systemic treatment algorithm for newly diagnosed advanced ovarian cancer. * FDA label indication. HGOC, high grade ovarian cancer; HRD, homologous recombination deficiency; IV, intravenous; NACT, neoadjuvant chemotherapy; PDS, primary debulking surgery; q3w, every 3 weeks.

The SOLO1 results placed maintenance olaparib as standard of care following platinum-based chemotherapy for women with newly diagnosed advanced high-grade serous EOC and a germline or somatic *BRCA1* and/or *BRCA2* mutation. New data from PAOLA-1, PRIMA, and VELIA have confirmed the benefit of PARP inhibitors for women with ovarian cancer and a BRCAm and have also proven benefit beyond BRCAm, most importantly in HRD-test positive subgroups. These four well-conducted studies have generated practice-changing data. However, deciding how to apply the recent data in clinical practice is challenging. The results may call for personalized medicine based on biomarker profiles and other factors. To aid clinical treatment decisions, we have proposed a systemic treatment algorithm for newly diagnosed advanced EOC in Figure 3. Alternative algorithms may use a different order of questions (e.g., considering HRD genomic instability earlier in the decision process).

The greatest magnitude of effect in all these studies was seen in patients with a BRCAm or a positive HRD test, regardless of BRCAm status. Biomarker testing is indicated in recent guidelines such as the National Comprehensive Cancer Network (NCCN) [36] and the European Society for Medical Oncology (ESMO) [37] recommendations. NCCN guidelines for newly diagnosed ovarian cancer recommend germline and/or somatic BRCAm testing and also state that HRD status in the absence of a germline and/or somatic BRCAm may provide information on the magnitude of benefit of PARP inhibitor therapy [36]. Testing of tumor samples to identify patients with both a germline and somatic BRCAm will allow identification of more patients that may benefit from PARP inhibitor therapy vs. germline testing alone [38].

Theoretically, HRD status can be assessed either by looking for the cause of HRD via assays that detect homologous recombination repair (HRR) gene mutations or by looking for the effect of HRD by evaluating the genomic instability caused by loss of HRR function [39]. The Myriad myChoice^®^ HRD assay detects both BRCAm and genomic instability and it is, so far, the only validated HRD test regarding PARP-inhibitor use in primary EOC. It assesses three independent measures of genomic instability, loss of heterozygosity (LOH), telomeric allelic imbalance, and large-scale state transitions across the genome, thereby increasing the prognostic power of the test. An alternative HRD assay is FoundationOne CDx™ (Foundation Medicine, Inc., Cambridge, MA, USA), which only detects LOH (via next-generation sequencing). There are limitations with commercially available HRD tests as some samples are returned with an “unknown” status and false negatives can occur. Although the greatest benefit is seen in BRCAm and HRD-test positive patients, HRD testing does not indicate if an individual patient will respond to a PARP inhibitor. Functional HRD testing may provide a better way to identify patients likely to benefit from PARP inhibitor therapy but needs to be validated in clinical studies [40].

PARP inhibitors are generally well tolerated. However, treatment decisions also need to take into account contraindications and the differences in tolerability profiles between PARP inhibitors, especially hematological events and the low occurrence of MDS and AML [41]. Cost effectiveness and regulatory access will also be a key consideration [42,43,44].

Given the approval of PARP inhibitors for first-line maintenance treatment, it is likely that a new profile of patients will emerge when their disease relapses. Such patients may or may not develop resistance to PARP inhibitor treatment and platinum-based chemotherapy rechallenge, and new treatment options will be required for those who become resistant. In addition, further assessment of outcomes from trials such as SOLO1 and PRIMA in terms of second line treatment outcomes may help us to better understand and define PARP inhibitor resistance. The Phase IIIb OReO study [45] is investigating maintenance olaparib rechallenge where patients have received one prior PARP inhibitor in a maintenance setting (this could be any PARP inhibitor, including olaparib) and were in complete or partial response to their most recent platinum-based chemotherapy regimen. Patients were heavily pre-treated, with 93% in the BRCAm cohort and 86% in the non-BRCAm cohort having received at least three prior lines of any chemotherapy. Recently reported primary results from OReO have shown that maintenance rechallenge with a PARP inhibitor (olaparib) provided PFS benefits to patients irrespective of BRCAm status: HR 0.57; 95% CI, 0.37–0.87; *p* = 0.022 in the BRCAm cohort and HR 0.43; 95% CI, 0.26–0.71; *p* = 0.002 in the non-BRCAm cohort [46]. A proportion of patients experienced clinically relevant long-term benefit with maintenance olaparib rechallenge.

Resistance to PARP inhibitors is an active area of research and reviewed in detail elsewhere [47,48,49]. Briefly, the main proposed mechanisms of resistance to PARP inhibitors include restoration of homologous recombination activity (e.g., by reactivation of BRCAm through secondary mutations), altered PARP expression, increased efflux of PARP inhibitors from cells, and stabilization of stalled replication forks (thereby reducing double-strand breaks). Several approaches to overcome these mechanisms of resistance (e.g., combining PARP inhibitors with agents such as immuno-oncology agents and cell cycle regulators such as WEE1), are being investigated [49].

## 5. Conclusions

The results of these four Phase III studies are practice changing, and recent PARP inhibitor approvals (olaparib plus bevacizumab; niraparib) in the newly diagnosed EOC setting offer new maintenance options for a broader patient population. However, with new treatment options come new challenges for clinical practice. It will be critical to identify newly diagnosed patients early for PARP inhibitor treatment by carrying out biomarker testing immediately at diagnosis (Figure 3). The authors propose this algorithm based on the results of the recent treatment trials presented in this manuscript, as well as available testing options. Although this algorithm would suggest a clearly defined and temporally separated process, the availability of tumor testing, the lack of uniform regulatory approvals for all drugs, and individual patient factors will play a role in its generalizability. Testing for both BRCAm and genomic instability will help inform treatment selection and the magnitude of PARP inhibitor benefit. Treatment decisions will depend on several factors, including biomarker profile, quality of life, contraindications and tolerability considerations, cost considerations and regulatory access, and physician and patient preference. Important areas for future research include improving HRD testing and exploring new options for patients who are HRD-negative and for those who become PARP inhibitor resistant.

## Figures and Tables

**Table 1:** Summary of key trial design and patient characteristics of Phase III trials of poly (ADP-ribose) polymerase (PARP) inhibitors for newly diagnosed advanced ovarian cancer.

ITT Population	SOLO1 [12] (GOG-3004) (NCT01844986)	PAOLA-1 [13] (ENGOT-ov25) (NCT02477644)	PRIMA [14] (ENGOT-ov26; GOG-3012) (NCT02655016)	VELIA [15] (GOG-3005; M13-694) (NCT02470585)
	*n* = 391	*n* = 806	*n* = 733	*n* = 1140
Key eligibility criteria	Stage III–IV disease *BRCA1* or *BRCA2* mutation Clinical CR or PR after first-line platinum-based chemotherapy Regardless of surgical status (upfront or interval surgery with or without residual macroscopic disease)	Stage III–IV disease NED or a clinical CR or PR after first-line platinum-taxane chemotherapy plus bevacizumab Regardless of surgical status (upfront or interval surgery with or without residual macroscopic disease)	Stage III disease with residual macroscopic disease after upfront surgery, inoperable disease, or NACT Any stage IV disease Clinical CR or PR (no measurable lesion >2 cm) and normal or >90% decrease in CA-125 after first-line platinum-based chemotherapy	Stage III–IV disease
Randomization	2:1 (within 8 weeks of last dose of chemotherapy)	2:1 (3–9 weeks after last dose of chemotherapy)	2:1 (within 12 weeks of last dose of chemotherapy)	1:1:1 (before the start of chemotherapy)
Stratification	Clinical response (clinical CR or PR)	First-line treatment outcome Tumor BRCA mutation status	Clinical response (clinical CR or PR) NACT HRD status	Timing of surgery and residual disease after primary surgery Paclitaxel schedule Disease stage Geographic region Germline BRCA mutation status
Intervention	Maintenance olaparib tablets (300 mg bid) vs. maintenance placebo	Maintenance olaparib tablets (300 mg bid) plus bevacizumab 15 mg/kg q3w vs. maintenance placebo plus bevacizumab 15 mg/kg q3w	Maintenance niraparib tablets (200 or 300 mg once daily individualized or fixed starting dose) vs. maintenance placebo	Carboplatin/paclitaxel * and veliparib (150 mg bid) followed by maintenance veliparib (300 mg bid for 2 weeks followed by 400 mg bid) (veliparib throughout) vs. Carboplatin/paclitaxel * and placebo followed by maintenance placebo (control) vs. Carboplatin/paclitaxel* and veliparib (150 mg bid) followed by maintenance placebo (veliparib combination only)
Duration of intervention	Until disease progression or up to 2 years; patients with ongoing PR at 2 years could continue receiving the intervention	Olaparib/placebo: until disease progression or up to 2 years or unacceptable toxicity Bevacizumab: 15 months total (including in combination with chemotherapy)	Until disease progression	36 × 3-week cycles (6 cycles of platinum-based chemotherapy and 30 cycles of maintenance therapy)
Primary endpoint	PFS (investigator assessed) in the overall population	PFS (investigator assessed) in the overall population, calculated from the end of chemotherapy	PFS (real-time blinded independent central review) in the overall population and HRD-test positive subgroup, calculated from the end of chemotherapy	PFS (investigator assessed) for the veliparib-throughout group in the BRCAm cohort, the HRD-test positive cohort, and overall ITT population (listed in order of testing hierarchy), calculated from the start of chemotherapy
Median follow-up, months	41	23	14	28

* Carboplatin AUC 6 mg/mL per min every 3 weeks; paclitaxel 175 mg/m^2^ every 3 weeks or 80 mg/m^2^ every week; AUC, area under the curve; bid, twice daily; CR, complete response; HRD, homologous recombination deficiency; ITT, intent to treat; NACT, neoadjuvant chemotherapy; NED, no evidence of disease; PFS, progression-free survival; PR, partial response; q3w, every 3 weeks.

**Table 2:** Poly (ADP-ribose) polymerase (PARP) inhibitors approved for the maintenance treatment of newly diagnosed ovarian cancer in the USA and Europe.

	FDA-Approved Indication	Approval Date	EMA-Approved Indication	Approval Date
Olaparib	Monotherapy [5] Maintenance treatment of adult patients with advanced epithelial ovarian, fallopian tube, or primary peritoneal cancer who have: ○ Complete or partial response to first-line platinum-based chemotherapy ○ Deleterious or suspected deleterious germline or somatic BRCA mutation Patients selected based on FDA-approved companion diagnostic	2018	Monotherapy [6] Maintenance treatment of adult patients with advanced (FIGO stages III and IV) high-grade epithelial ovarian, fallopian tube, or primary peritoneal cancer who have: ○ Complete or partial response to first-line platinum-based chemotherapy ○ Germline and/or somatic *BRCA1/2*-mutation	2019
	Combination with bevacizumab [5] Maintenance treatment of adult patients with advanced epithelial ovarian, fallopian tube, or primary peritoneal cancer who have: ○ Complete or partial response to first-line platinum-based chemotherapy ○ Cancer associated with HRD-positive status defined by either a deleterious or suspected deleterious BRCA mutation, and/or genomic instability Patients selected based on FDA-approved companion diagnostic	2020	Combination with bevacizumab [6] Maintenance treatment of adult patients with advanced (FIGO stages III and IV) high-grade epithelial ovarian, fallopian tube, or primary peritoneal cancer who have: ○ Complete or partial response to first-line platinum-based chemotherapy in combination with bevacizumab ○ Cancer associated with HRD-positive status defined by either *BRCA1/2* mutation and/or genomic instability	2020
Niraparib	Monotherapy [7] Maintenance treatment of adult patients with advanced epithelial ovarian, fallopian tube, or primary peritoneal cancer who have: ○ Complete or partial response to first-line platinum-based chemotherapy	2020	Monotherapy [8] Maintenance treatment of adult patients with advanced (FIGO stages III and IV) high-grade epithelial ovarian, fallopian tube, or primary peritoneal cancer who have: ○ Complete or partial response to first-line platinum-based chemotherapy	2020

EMA, European Medicines Agency; FDA, US Food and Drug Administration; FIGO, International Federation of Gynecology and Obstetrics; HRD, homologous recombination deficiency.

**Table 3:** Summary of reported adverse events in the Phase III SOLO1, PAOLA-1, PRIMA, and VELIA trials. (**A**) SOLO1 [12], (**B**) PAOLA-1 [13], (**C**) PRIMA [7], (**D**) VELIA (maintenance phase) [15].

**(A)**		
**Olaparib (*n* = 260) vs. Placebo (*n* = 130)**		
	**Any Grade**	**Grade 3 or 4**
Hematological AEs, %		
Anemia *	39 vs. 10	22 vs. 2
Neutropenia ^†^	23 vs. 12	9 vs. 5
Thrombocytopenia ^‡^	11 vs. 4	1 vs. 2
Most common non-hematological AEs, %		
Nausea	77 vs. 38	1 vs. 0
Fatigue/asthenia	63 vs. 42	4 vs. 2
Vomiting	40 vs. 15	<1 vs. 1
Diarrhea	34 vs. 25	3 vs. 0
Constipation	28 vs. 19	0
Dysgeusia	26 vs. 4	0
Arthalgia	25 vs. 27	0
Abdominal pain	25 vs. 19	2 vs. 1
**(B)**		
**Olaparib Plus Bevacizumab (*n* = 535) vs. Placebo Plus Bevacizumab (*n* = 267)**		
	**Any Grade**	**Grade 3 or 4**
Hematological AEs, %		
Anemia *	41 vs. 10	17 vs. <1
Lymphopenia ^§^	24 vs. 9	7 vs. 1
Neutropenia ^†^	18 vs. 16	6 vs. 3
Leukopenia ^¶^	18 vs. 10	2 vs. 1
Thrombocytopenia ^‡^	8 vs. 3	2 vs. <1
Most common non-hematological AEs, %		
Fatigue/asthenia	53 vs. 32	5 vs. 1
Nausea	53 vs. 22	2 vs. 1
Hypertension	46 vs. 60	19 vs. 30
Arthalgia	22 vs. 24	1 vs. 1
Vomiting	22 vs. 11	1 vs. 2
Abdominal pain	19 vs. 20	1 vs. 2
Diarrhea	18 vs. 17	2 vs. 2
Urinary tract infection	15 vs. 10	<1 vs. <1
**(C)**		
**Niraparib (*n* = 484) vs. Placebo (*n* = 244)**		
	**Any Grade**	**Grade 3 or 4**
Hematological AEs, %		
Thrombocytopenia **	66 vs. 5	39 vs. <1
Anemia **	64 vs. 18	31 vs. 2
Neutropenia ^†^^†^	42 vs. 8	21 vs. 1
Leukopenia ^‡^^‡^	28 vs. 9	5 vs. <1
Most common non-hematological AEs, %		
Nausea	57 vs. 28	1 vs. 1
Fatigue **	51 vs. 41	3 vs. 1
Constipation **	40 vs. 20	1 vs. 0.4
Musculoskeletal pain **	39 vs. 38	1 vs. 0
Headache	26 vs. 15	<1 vs. 0
Insomnia	25 vs. 15	1 vs. <1
Dyspnea **	22 vs. 13	<1 vs. 1
Vomiting	22 vs. 12	1 vs. 1
**(D)**		
**Veliparib-Throughout (*n* = 310) vs. Placebo (*n* = 311)**		
	**Any Grade**	**Grade 3 or 4**
Hematological AEs, %		
Thrombocytopenia	20 vs. 5	7 vs. <1
Anemia	17 vs. 10	7 vs. 1
Neutropenia	17 vs. 12	5 vs. 4
Leukopenia	10 vs. 5	1 vs. 1
Most common non-hematological AEs, %		
Nausea	56 vs. 24	5 vs. 1
Vomiting	34 vs. 12	2 vs. <1
Fatigue	23 vs. 18	6 vs. 1
Diarrhea	19 vs. 18	<1 vs. <1
Abdominal pain	18 vs. 18	3 vs. 1
Arthalgia	16 vs. 20	<1 vs. <1
Peripheral sensory neuropathy	16 vs. 18	<1 vs. <1
Insomnia	13 vs. 10	1 vs. 0

It is important to note that cross-trial comparisons should not be made due to differences in patient populations and trial methods across each of the studies. Data are shown for hematological adverse events that occurred in at least 10% of the patients in either trial group, except for thrombocytopenia in PAOLA-1 which occurred in less than 10% of patients in either trial group. However, data are included to provide a full profile of hematological adverse events. PRIMA data are from the FDA prescribing information as this reports grouped terms for these AEs, similar to the other studies. Green highlighting shows adverse events that occur at either the same or a lower incidence in the experimental vs. control arm. * Data include patients with anemia, decreased hemoglobin level, decreased hematocrit, decreased red-cell count, erythropenia, macrocytic anemia, normochromic anemia, normochromic normocytic anemia, or normocytic anemia. ^†^ Data include patients with neutropenia, febrile neutropenia, neutropenic sepsis, neutropenic infection, decreased neutrophil count, idiopathic neutropenia, granulocytopenia, decreased granulocyte count, or agranulocytosis. ^‡^ Data include patients with thrombocytopenia, decreased platelet production, decreased platelet count, or decreased plateletcrit. ^§^ Data include patients with decreased lymphocyte count, lymphopenia, decreased B-lymphocyte count, or decreased T-lymphocyte count. ^¶^ Data include patients with leukopenia or decreased white-cell count. ** Grouped preferred terms. ^††^ Data include patients with neutropenia, neutropenic infection, neutropenic sepsis, or febrile neutropenia. ^‡^^‡^ Data include patients with leukopenia, decreased lymphocyte count, lymphopenia, or decreased white blood cell count. AE: Adverse event. SOLO1 [12] adapted from *N. Engl. J. Med.*, Moore, K. et al. Maintenance olaparib in patients with newly diagnosed ad-vanced ovarian cancer, Vol. 379, p2504. Copyright © (2018) Massachusetts Medical Society. Reprinted with permission from Massachusetts Medical Society (SOLO1 adverse-event data). PAOLA-1 [13] from *N. Engl. J. Med.*, Ray-Coquard, I. et al. Olaparib plus bevacizumab as first-line maintenance in ovarian cancer, Vol. 381, p2426. Copyright © (2019) Mas-sachusetts Medical Society. Reprinted with permission from Massachusetts Medical Society (PAOLA-1 adverse event data). PRIMA [7] from the ZEJULA™ (Niraparib) prescribing information. Reproduced with permission from the U.S. Food and Drug Administration (PRIMA adverse-event data). VELIA [15] From *N. Engl. J. Med.*, Coleman, R.L. et al. Velip-arib with first-line chemotherapy and as maintenance therapy in ovarian cancer, Vol. 381, Suppl. Appendix p17. Copy-right © (2019) Massachusetts Medical Society. Reprinted with permission from Massachusetts Medical Society (VELIA adverse-event data).
